# Compendium of Midwifery for Midwives in the Grand Duchy of Baden

**Published:** 1845-01

**Authors:** 


### ART. VI.

1. *Lehrbuch der Geburtshülfe für die Hebammen im Grossherzogthume Baden.* Von FRANZ CARL NAEGELE, Grossherzoglich Badischem Geheimerathe, ordentl. offentl. Professor der Medizin und Geburtshülfe, Direktor der Entbingdungs-anstalt zu Heidelberg, &c. &c.—*Heidelberg*, 1844.

*Compendium of Midwifery for Midwives in the Grand Duchy of Baden.* By F. C. NAEGELE, M.D., Professor of Medicine and Midwifery, and Director of the Lying-in Hospital at Heidelberg. Sixth Edition.—*Heidelberg*, 1844. 8vo, pp. 408.

2. *Lehrbuch der Geburtshülfe.* Von Dr. HERMANN FRANZ NAEGELE, ausserordentlichem Prof. der Medicine an der Universität Heidelberg. Erster Thiel—*Physiologie und Diätetik der Geburt.—Mainz*, 1843.

*Compendium of Midwifery.* By Dr. H. F. NAEGELE, Professor extraordinary of Medicin at the University of Heidelberg. Part First—*Physiology and Treatment of Labour. — Mayence*, 1843. 8vo, pp. 390.

As the first edition of Professor Naegele's 'Hebammen-buch,' was published before the commencement of this Journal, a review of it has not appeared in our pages; but the value of the work, the numerous editions it has gone through, and above all the high name of its excellent and distinguished author, make it a matter of duty not less than of pleasure to take the present opportunity of bringing it under the notice of our readers; the more so as we are enabled to combine under the same review the first part of a Manual of Midwifery by his son, Professor Naegele, jun.

The value of an Hebammen-buch, or work on midwifery intended for the use of midwives, is somewhat doubtful in the estimation of a considerable portion of the profession in England; although for our own parts we have ever thought that a simple and concise exposition of those points in practical midwifery with which it is necessary that a midwife should be acquainted, is not only a great desideratum in this country, inasmuch as it would prove highly valuable to a numerous class of female practitioners who are at present without any work upon the subject which is adapted to them, but is also, in our opinion, by no means unworthy the pen of a London accoucheur of eminence in his profession.

Although we are well aware that we could point out many midwives, who are thoroughly acquainted with their profession, nay perhaps even more so than many of their *brother* practitioners, it must nevertheless be confessed that the majority, especially of those in the country, are grossly ignorant and illiterate; and that few, except the better sort who have been enabled to receive instructions in London, possess the proper amount of knowledge upon the subject. No laws or enactments exist in this country for the better regulation of midwives, for requiring and ensuring certain qualifications as regards respectability, general education, as well as having received practical instructions in midwifery from a properly authorised professor. No law in England professes to exercise the slightest jurisdiction on this important subject; and as this is a great evil, we trust that means will be found to abate it in the forthcoming Medical Bill.

In Germany the case is very different: intelligent women, possessing the necessary degree of education, are selected from the different towns and villages, and sent to the lying-in hospital of the nearest university for instruction, which they receive from the Professor himself, and attend a certain number of labours there; they are then strictly catechised as to their proficiency before receiving their license to practise. At certain periods also, viz., every one or two years, all the midwives of the district are summoned before the Professor to show that they possess the necessary knowledge of their business, and in this way the whole body of female practitioners, throughout an extensive district, are kept under the immediate supervision of the authorities, and their fitness for practising midwifery strictly tested.

We cannot agree with Professor Naegele, jun., when he states that it is "unnecessary for an accoucheur to attend natural labours, as being a branch of practice essentially belonging to midwives; and although to a scientific practitioner the observing such cases must ever be a matter of interest, yet it would be quite inadmissible for him to make it a source of emolument." (§ 7.) In all great cities, and among the better classes, the management of natural labour will always in great measure devolve on male practitioners, for it is well known that in case of danger they, after all, must be the authorities to whom recourse will be had for assistance in time of need; and as moreover these patients, from their means and station in society, are enabled to command professional attendance of the highest rank, they naturally prefer committing the entire management of their labour to practitioners of this class, to trusting to the discrimination, experience, and perhaps honesty of a midwife for deciding upon the necessity of calling them in. With practitioners of a lower grade, (we allude to the general practitioners,) the same holds good in preferring them to midwives for taking charge of cases of natural labour: firstly because their medical education has been (and therefore ought to have rendered them) vastly superior; and 2dly, because they will therefore be better able to distinguish and appreciate danger in its earliest stages.

In the work of Professor Naegele, sen., we of course find no allusion to the literature of the subject; it is an elementary work of the simplest kind; the arrangement is admirable, the expositions are clear and comprehensive. It commences with a short view of the structure of the human frame, its various organs, and their functions. The parts concerned in pregnancy, labour, &c., are described more fully, but yet in a manner quite intelligible to the class of readers for whom the book is ostensibly intended. The whole work is divided into two parts: the 1st, comprising pregnancy, labour, and the puerperal state under healthy circumstances; the 2d, labour, the puerperal state, and pregnancy under abnormal circumstances. In the latter division he makes use of the same arrangement of Dystocia, which he first described in his 'Erfahr. und Abhandl." 1811, and which subsequent years and experience have proved to be one of the best and most valuable known. He divides the subject of dystocia or faulty labour under two heads: viz., 1, "faulty labours from difficulty or impossibility in completing labour by the natural powers;" and 2, "faulty labours without difficulty in the process."

It is not very easy to give the word "*fehlerhaft*" its full signification in English; we think that "*faulty*" expresses it as perfectly as "*vicieux*" which the French apply to it; perhaps *abnormal* would be better, but in reference to labour, the Greek prefix δυς, in the word dystocia, appears to answer the purpose completely, and has been adopted by general consent.

Under the first head of dystocia Professor Naegele brings the six following species:

1. Dystocia from malposition of the child.
2. ,, faulty size and form of the child,
3. ,, ,, condition of the parts which belong to the child.
4. ,, ,, ,, hard passages (pelvis.)
5. ,, ,, ,, soft passages.
6. ,, ,, ,, expelling powers.

Under the second head, where labour is rendered unfavorable or faulty, without any difficulty or obstruction to its progress he places the four following species:

1. Dystocia from the labour following too rapid a course.
2. ,, prolapsus or otherwise faulty condition of the umbilical cord.
3. ,, other faulty circumstances which render labour dangerous, (convulsions, hemorrhages, &c.)
4. ,, faulty separation and expulsion of the placenta, hemorrhage after labour, and inversion of the uterus.

This arrangement has been adopted by Dr. Rigby in his 'System of Midwifery,' of which we made favorable mention in vol. XII, p. 461.

The description of the placenta by Professor Naegele, jun., is very meager and imperfect. He quotes the recent observations of Professor

E. H. Weber, where he shows that the vessels which pass through the placental decidua, are merely a continuation of the lining membrane of the uterine vessels; receiving also a covering from the soft substance of the decidua. Beyond its bare mention, the placental decidua is entirely omitted, not a word about its curling arteries, or the manner in which the maternal blood circulates through the placenta, nor any description of the orifices which are seen in the sulci between the cotyledons of the placenta, and which mark where the curling arteries of the decidua had been torn through when the placenta was detached from the uterus. Dr. W. Hunter's anatomical description of the human gravid uterus, translated by Froriep is mentioned, it is true, and so indeed is Lobstein's admirable 'Essai sur la Nutrition du Fœtus,' but the author has not in our opinion availed himself sufficiently of their valuable contents; still less of J. Hunter's observations on this subject, which, as also the more recent ones of Dr. Reid, he has omitted altogether; indeed we regret that the whole description of the ovum has been discussed so briefly.

In describing the conformation of the head of a full-grown fetus, Professor Naegele, jun., gives the following judicious remarks:

"It is of great importance, as regards labour, that the head of a child is capable of undergoing such alterations in its form and size, as to adapt it for passing through the pelvis. As the bones which form the arch of the cranium are moveable at their attachments to each other, their edges are not only capable of greater approximation, but may be made to overlap each other, an arrangement the value of which we see in difficult labours, from misproportion between the head and pelvis. The size of the head, especially in the direction of its straight or transverse diameters can thus be diminished several (six, nine,) lines. The basis cranii alone does not admit of any alteration, for the different bones of this part, and the cartilages which unite them form a solid whole in the full-grown fetus.

"The capability of the head for undergoing this change in its configuration does not only depend on the breadth and elasticity of its sutures, but also on the more or less yielding and flexible nature of the bones themselves. In both respects the child's head presents considerable varieties, and is therefore equally variable in the degree of reduction of size which it is capable of obtaining." (§ 126.)

An important fact can scarcely be noticed too frequently, particularly when it is set before the reader in a clear and intelligible manner. The alteration which the head of a full-grown living fetus is capable of undergoing during its progress through the pelvis and external passages, and of adapting its shape by the pressure of the surrounding parts, so as to present the least possible resistance, is one of the most remarkable, and, we may add, beautiful features in the mechanism of parturition. Without this provision, labour would frequently be attended with the most serious and fatal difficulties, owing to the firm unyielding structure of the human pelvis; whereas in the inferior animals a variety of not less interesting changes take place in the different symphyses of the pelvis shortly before and during labour, and enable it to undergo a considerable degree of dilatation, and thus attain the same object on the part of the maternal passages which is gained by yielding and overlapping of the cranial bones of the human fetus.

Professor Naegele, jun., in all the practical details connected with the physiology and treatment of natural labour, has not only strictly followed the same arrangement as that which his father has adopted, but in many cases has embodied, with little or no modification, the paragraphs of the 'Hebammenbuch' into his own work.

To one who is unacquainted with the condensed and laboured style of Professor Naegele, sen., where every sentence has undergone a degree of careful consideration and frequent correction and remodelling, to an extent which can scarcely be credited, it might seem hardly justifiable even for a son to make so free with a work as in all essential particulars to transpose a large portion of it into his own; but uniting in the same individual the relation of parent and teacher, it is not more than natural that he should look upon the highly-finished, and we had almost said perfect, descriptions of his father as incapable of further improvement; still we should have preferred seeing them more strictly quoted and acknowledged; and, after all, the brief manner in which the various subjects are necessarily treated in the 'Hebammen-buch,' is hardly sufficient for a work of the nature which the other professes to be.

In the rules of conduct for a woman during her pregnancy, Professor Naegele, gives the following important axiom: "A woman with child should as far as possible essentially maintain the same mode of living to which she has been previously accustomed, and which had agreed with her before her pregnancy, and only avoid that degree of excess, and everything which may be injurious to her as a pregnant woman." (§ 217.) The old and mischievous notions that a woman must take more than her ordinary amount of food during pregnancy, and less than her ordinary amount of exericse; that she should indulge every slothful feeling, and gratify every morbid whim and longing, which are but too frequently the offsprings of an uninformed and unemployed mind, are fortunately falling into well-merited disgrace and oblivion among the better-educated classes of this country; and with the cultivated portion of our German friends, it is much the same, whereas in the more remote districts in Germany, in a large part of France, and even more remarkably in the Baltic provinces of Russia, all these superstitious absurdities are still in vogue even amidst the higher orders.

We quite agree with Professor Naegele, in saying that "the action of the abdominal muscles is only for the purpose of supporting the contractions of the uterus, and contributes much less to the expulsion of the child; so that labour is even capable of being completed without any assistance from the abdominal muscles." (§ 283.) We differ from Professor Naegele, jun., in saying that the abdominal muscles and *diaphragm* assist the uterine contractions, for there is no reason to suppose that the diaphragm takes any part in the bearing-down efforts which characterize the expelling pains of labour, beyond effecting a deep inspiration, immediately before the effort is made, in order to reduce the capacity of the abdominal cavity still further. We cannot imagine that muscles of inspiration and expiration can ever act *simultaneously*, and therefore against each other; and if we watch the phenomena which immediately precede and attend a powerful effort, we shall find that the first act is to take a deep inspiration and inflate the chest, the next is to close the glottis by the arytenoid muscles, in order to prevent the air escaping from the lungs, and now that the abdominal cavity is diminished to the utmost, and the thorax fixed in its state of inflation, the effort is made under the most favorable circumstances for exerting the muscles of the trunk, particularly those of the abdomen. The diaphragm ceases to contract during the effort, as is seen by the sudden and forcible expiration which follows the effort, and which could not take place if the diaphragm were still acting. We see the same also when a person is prevented closing the glottis by laughter or other involuntary sounds ; the power of making a muscular effort is greatly reduced, and the first warning that a violent exertion is about to be made, is the deep inspiration and closed lips which betoken the above-mentioned preparations for that purpose.

Professor Naegele divides the process of labour into five stages, an arrangement which is generally made use of in Germany, and we think it in many respects preferable to the more simple division into three stages, as adopted by the English authors, from its enabling us to give a better description of the whole process. We have heard it used by Dr. Rigby when lecturing on this subject at St. Bartholomew's, who agrees with us in this opinion. Professor Naegele's description of the phenomena which take place during these five stages of labour, forms the most masterly exposition of the whole process we have ever seen ; for simple, concise, and yet comprehensive description we could almost pronounce it unequalled.

" *The first stage*, which might properly be called the warning stage, commences with the first preceptible contractions of the uterus, and in primiparæ last until the os uteri opens, but in multiparæ, in whom the os uteri is slightly open during the latter part of pregnancy, until it begins to dilate. These first contractions of the uterus, which are called the *precursory pains*, produce a sensation to the patient as if the abdomen over its whole extent were equally compressed ; after a short time it again ceases. This is also accompanied by a heavy uncomfortable feel of dragging about the pelvis, especially the sacrum, not amounting to actual pain. If the hand be laid upon the abdomen at this moment, the uterus feels distinctly hard and tense, and when the above sensation ceases, it becomes soft. In primiparæ the precursory pains continue for a longer, and in multiparæ for a shorter time before labour. Sometimes they last only twelve, eighteen, or twenty-four hours, sometimes several days. They usually come on towards evening, and go off during the night. In this stage, the duration of which is so variable, a copious secretion of mucus takes place from the vagina, and there are frequent calls to pass water. The patient feels restless and anxious ; the abdomen subsides still more, and the inferior segment of the uterus, especially in primiparæ, sinks still deeper into the cavity of the pelvis. The os uteri nevertheless is frequently difficult to find at this moment, partly because it is turned quite backwards, viz., towards the sacrum, but chiefly because it is very small, and frequently presents a little depression not larger than a lentil. On the other hand in multiparæ, the thick cushiony os uteri is open to such an extent that the membranes, and through them the presenting parts of the child may be felt. In many cases, and especially in irritable habits, or in consequence of having caught cold, particularly in primiparæ, the precursory pains are so severe as to be mistaken for those of actual labour. The midwife, however, will be able to decide, from the dilatation or non-dilatation of the os uteri, whether labour has really commenced or not." (§ 236.)

" *The second stage* of labour begins with the opening of the os uteri, and in multiparæ with its dilatation. The contractions are now attended with pain, and return more frequently and regularly. The pains go from the loins towards the pubes, but are most severe in the sacrum. The os uteri gradually dilates more and more. During each pain the liquor amnii which is contained in the membranes, is pressed against the dilated os uteri, and the membranes thus distended are pushed through it during a pain in an hemispherical or egg-shaped form. When the pain goes off, the bag of membranes becomes again soft and relaxed, and the presenting head may be again felt through the membranes. The inferior segment of the uterus and the os uteri, which during the pain were hard, again become soft and yielding as they were before the pain commenced. If the os uteri be dilated to the extent of four fingers, and the bag of membranes, which increases in proportion, projects deep into the pelvic cavity, it may be expected to burst during the next pain or so. Because the pains during this stage of labour dilate the os uteri, and thus prepare it for the passage of the child, they are called the *preparatory* pains. They are usually more intolerable to the patient than those during the further progress of the labour, and this chiefly arises from their appearing to her to be of no use, and giving her no proof that labour has advanced, and that she will soon be relieved. The mucus which escapes from the vagina at the beginning of this stage, and which is perceived upon the finger after examination, is mixed with streaks of blood; this is called by midwives '*a show*,' because it is a sign that the os uteri has actually dilated, and that labour has commenced." (§ 237.)

"*The third stage* commences with the discharge of the waters. The pains usually abate somewhat immediately after this, but then return more frequently, stronger, more piercing, and affect the rest of the body. The patient begins to tremble, and can usually neither stand nor sit. The face becomes red and hot. Perspiration breaks out over the whole body. An irresistible impulse to strain accompanies every pain, viz., to support the uterine contractions by those of the abdominal muscles. Frequent desire to relieve the bladder and the rectum. Increasing impatience and frequent fretting and complaining of the pain which is chiefly in the sacrum. Frequent painful cramp in the thighs and calves of legs.

"After the discharge of the liquor amnii, the uterus contracts closer upon the child, and its inferior segment invests the head more tightly, and embraces it more firmly. Hence the cranial bones begin to overlap, the scalp becomes corrugated, and at this spot the caput succedaneum forms during the further progress of labour. The pains of this stage are denominated the *peculiar* pains of labour." (§ 238.)

"*The fourth stage* begins when the head becomes visible between the labia, and ends with the birth of the child. The head presses against the os externum during every pain, forces the labia asunder, and a portion of it becomes visible externally. It presses also upon the perineum, drives it downwards in the form of a ball, and distends it in all directions, from behind forwards, from one side to the other, so that this part which from behind forwards was scarcely the breadth of two fingers, at this stage of the labour becomes four and even more, and therefore gradually grows thinner, and is in danger of giving way. The anus is strongly dilated, and the detruded anterior wall of the rectum becomes pushed out and visible. As the pain goes off the head retires again, usually much slower than it was pushed forwards by the pain, and the violent distension and stretching of the parts again abates. Liquor amnii escapes at times, and chiefly at the beginning and end of a pain; rarely during the pain, or during the intervals. As this stage of labour advances a larger portion of the head becomes visible, and at length advances so far through the labia that nearly the greatest circumference which it presents is now surrounded by the os externum; it now either passes quickly through, or (which is more frequently the case in primiparæ) it does not recede as the pain goes off, but remains stationary, and with the following pain, if this be strong enough, presses with its greatest circumference through the immensely distended os externum.

"In primiparæ, if the head when nearly expelled recedes somewhat as the pain subsides, it is attended with a slight discharge of blood.

"The pains of this stage are still more severe, painful, and enduring; return after a shorter interval, and take a far greater effect upon the patient than those of the previous stage. Their severity increases so much the more from the additional suffering arising from the continually increasing distension of the external parts. They convulse the whole frame, and have hence been called the *Dolores conquassantes*.* The bearing down becomes more continued, and there is not unfre-

* We have given the received Latin name in preference.

quently vomiting. The patient quivers and trembles all over. Her face is flushed, and with the rest of the body is bathed in perspiration. Her looks are staring and wild; the features alter so much that she can scarcely be recognized. Her impatience rises to its maximum. Loud crying and wailing, and frequently expressions which, even with sensible high-principled women, border closely upon insanity: everything denotes the violent manner in which both body and mind are affected.

"The moment the head has passed, the pains cease, and the patient finds herself in a state of the most delightful calm. After an interval of more or less duration, fresh pains, but far less severe come on, by which the shoulders are pushed through, after which the rest of the child usually follows quickly, and the remainder of the liquor amnii escapes. A little blood or a few coagula usually come away with the liquor amnii, or immediately afterwards. The contracted uterus, unless there be twins, feels now like a ball of the size of a large child's head. After the birth of the child a violent shivering usually follows." (§ 240.)

"The *fifth stage* commences immediately after the birth of the child, and ends with the expulsion of the placenta.

"Immediately after the birth of the child the smooth inner surface of the placenta may generally be felt bulging through the os uteri; a proof that the placenta is usually detached from the uterus immediately after the birth of the child, and lies free within it. In a quarter of an hour, more or less, sometimes half an hour or even three-quarters of an hour, fresh uterine contractions came on, which are usually not particularly painful, and which are called the afterbirth pains or dolores cruenti. A moderate quantity of blood sometimes escapes during these pains. By means of these afterbirth pains the placenta is pushed down through the os uteri, with its inner or smooth surface foremost, into the vagina, and together with the inverted membranes, is ultimately expelled by the joint efforts of the uterus and vagina." (§ 240.)

Professor Naegele, jun. considers that the mean duration of labour is between six and twelve hours, and quotes the valuable results of Dr. Collins's practice; where of 15,850 labours, 15,084 were completed within twelve hours, and of these the greater part, viz. 10,987 within from one to four hours. (§ 250.)

To his subdivision of healthy labours, Professor Naegele, sen., premises the following admirable remarks; we recommend them strongly to the attention of our junior readers, as a proof that no labour, however natural, can be uninteresting, but that even the most natural and favorable cases will present to the diligent observer much that is valuable and instructive.

"The function of parturition possesses various peculiarities, which, apart from its peculiar object, distinguish it from all other functions. Thus, for instance, while the other functions, as long as the individual is healthy, proceed without the slightest pain, but rather a sensation of comfort, parturition, even in a state of health, is attended with pain and efforts, and exertion of strength. Apart from all this, the very important peculiarity occurs in parturition, that nature follows less accurately a certain course, and permits of greater deviations from the ordinary process than in the other functions without its object, viz. the safe expulsion of the fetus, being interfered with. No two labours are exactly alike, even where the result in other respects is equally favorable; and all experienced practitioners agree in saying, that almost every labour presents some peculiarity, and some more or less striking deviation from the ordinary course of this process. Thus, for instance, the duration of the whole labour, or of its different stages is very different. The greatest variety prevails in the severity of the pains, their strength, duration, frequency, and influence on the rest of the system. How variable is the quantity of liquor amnii and of blood discharged during labour, as also the weight of the full-grown fetus (five to nine pounds!) But one of the most remarkable variations is the position which the child presents during labour. For although the presentation of the cranial portion of the head is the most common, the child occasionally presents with the face, more frequently with the nates or feet.

"But all such deviations from the ordinary course, inasmuch as neither mother nor child is exposed to any particular injury or danger, are by no means to be considered as morbid or faulty, but as modifications of the healthy state, such as are observed in the other functions of the human body, only in a less degree." (§ 250.)

"When the axis of the child's body corresponds with that of the uterus, it can be born by the natural powers without danger or injury, supposing that the other requisites for labour are in a healthy state. This can occur in two ways, either the child presents with its upper end or head, or with the lower end or nates." (§ 251.)

Professor Naegele arranges the natural presentations under four heads:

1. Cranial presentations.
2. Face presentations.
3. Nates presentations.
4. Foot presentations.

In 100 labours the average is, that the cranium presents about ninety-three or ninety-four times; the nates and feet about four times, but the face does not occur more than once in 200 cases.

The signs of a natural presentation, and the enumeration of what the finger will detect during examination of the head in the first position, are given with that lucid preciseness which characterize the descriptions of this distinguished teacher.

"That the child is in a favorable position for labour, viz. with its long axis parallel with that of the uterus, may be inferred, towards the end of pregnancy or commencement of labour, from the following symptoms: 1st, when the abdomen is pointed forwards, equally distended, and not too broad at the sides, and shows no marked inequalities; 2d, if the patient feel the motion of the child only at the side, whether it be the right or left; and, 3d, if, on internal examination, we feel a large, round, spherical body presenting. That the presenting part is the head, viz. the cranium, is known by its being uniformly convex and hard. The species of the cranial position will be ascertained from the direction of the sutures and position of the fontanelles. The diagnosis will be rendered difficult by the following circumstances, viz. the head being high up in the pelvis, and moveable; much liquor amnii between the head and membranes; the bladder of membranes remaining tense between the pains; swelling of the cranial integuments; softness of the cranial bones, which sometimes feel like tinsel or parchment, or otherwise faulty development of them; numerous false sutures, &c." (§ 254.)

The head usually presents, during labour, in one of two ways at the superior aperture of the pelvis: viz.

"1, With the right parietal bone (as the part which is deepest in the pelvis) foremost, and small fontanelle towards the left, and more or less forwards; and, 2, with the left parietal bone foremost, the small fontanelle in exactly the contrary direction, viz. to the right, and more or less backwards." (§ 255.)

The first of these two positions is well known to be that which of all others occur most frequently. This is a well-established fact, recognized by all authors and teachers of midwifery since the time of Matthias Saxtorph and Solayres de Renhac, the teacher of the celebrated Baudelocque,* (1771,) who first pointed out the real position of the head in the pelvis at the commencement of labour. The relative frequency of these two positions of the head is between 1 and 1½ to 2. Thus, in 100

* De diverso partu ob diversam capitis ad pelvim relationem mutuam; Hafniæ, 1771. De partu viribus maternis absoluto; Paris, 1771.

cases of cranial presentation, calculated from a great number, the average number in the first position will be about 70, and of the second about 30. According to Professor Naegele, jun., of 3491 cases of cranial presentation which were accurately noted at the Lying-in Hospital of Heidelberg, 2262 were in the first, and 1217 in the second position.

Professor Naegele, senior's description of the manner in which the head presents and passes through the pelvis and soft parts, when in the first position, is by far the most perfect and complete which we are acquainted with; we quote it in order that our junior readers may profit by it, as we question if such a description of natural labour exists in this language:

"If we examine quite at the beginning of labour, as soon as the os uteri is sufficiently open to let the finger pass, the point of it touches upon a suture, which crosses the os uteri transversely. This is the sagittal suture, and the spot which the finger touches is about the middle of it. If we move the point of the finger along this suture, towards the left, it comes to a place where it seemingly divides into two sutures, from its meeting with two other sutures. These are the two branches of the lambdoidal suture, and the spot where they meet the sagittal suture is the small or posterior fontanelle. If we move the finger from that part of the sagittal suture which corresponds to the os uteri, along it in the contrary direction, we come to a quadrangular spot, without bone, in which four sutures terminate; this is the great or anterior fontanelle. If we bring the finger from that part of the sagittal suture which corresponds to the os uteri, viz. when the os uteri is sufficiently open and soft, in a direction straight forwards, it will come to a projecting conical-formed part of the head, viz. the tuber of the right parietal bone. If the anterior lip of the os uteri does not permit the finger to reach so far forwards, we shall be able to distinguish the parietal protuberance through the inferior segment of the uterus.

"The position of the head at the beginning of labour in the first cranial position is therefore as follows: The vertex is turned towards the sacrum, viz. the middle position of the sagittal suture to the body of the first or second sacral vertebra (according as the head is high or low in the pelvis); the occipital fontanelle is directed to the left, and somewhat forwards, the anterior fontanelle to the right, and somewhat backwards; the right parietal bone is the part which is deepest, and its protuberance is nearly in the middle of the pelvis. The head, therefore, presents during labour at the upper aperture of the pelvis, in a direction which is oblique, both as to its axis and transverse diameter." (§ 258.)

"During the further progress of labour, viz. while the head is pressing through the superior aperture and gradually sinking into the cavity of the pelvis, the two fontanelles frequently are found at an equal height with each other; sometimes the anterior, but more frequently the occipital fontanelle is lowest as the head descends.

"If the head has passed through the upper aperture of the pelvis, with the largest circumference which it presents to it, and approaches to the lower portion of the pelvic cavity, the two fontanelles are usually found to be equally advanced, the posterior turned towards the left foramen ovale, the anterior one to the right sacro-ischiatic notch. The antero-posterior diameter of the head corresponds to the right oblique diameter of the pelvic cavity." (§ 259.)

"As the head becomes pressed against the perineum and os externum, towards the end of the third stage, by the continued action of the pains, and begins to push down the one and to dilate the other, so as to be visible between the labia during a pain, it is the *superior and posterior quarter* of the right parietal bone which corresponds to the os externum, and the right branch of the lambdoidal suture may now be felt running parallel with the descending ramus of the left os pubis.

"The superior and posterior quarter of the right parietal bone is that part of the cranium which first enters the vagina, and the head maintains this oblique direction during its further advance through the os externum, even at the point of its expulsion; if the process be sufficiently slow, careful observation will show that the posterior fontanelle is usually still turned somewhat to the left. On examination, we shall find the right branch of the lambdoidal suture higher, or nearer to the apex of the pelvic arch than the left one.

"As soon as the head is freed from the resistance which it had experienced from the soft parts when passing through the os externum, it resumes its former oblique position, even if it had not maintained it during its expulsion, viz. the face turns backwards and to the right." (§ 260.)

We would fain quote every paragraph on this important and deeply interesting subject, but our limits render this quite impossible; we must therefore proceed to Professor Naegele's description of the mechanism of parturition where the head presents in the second cranial position, and merely content ourselves with saying, that those who wish to gain true and correct views of natural labour, should study these masterly expositions of it at the bedside of their patients.

In the second cranial position, the head, at the commencement of labour, is just as oblique, as regards the transverse diameter and axis of the pelvis, as in the first, with the difference only that the situation of the fontanelles is the reverse of what it was in that position, viz. the anterior fontanelle is now where the posterior one was in the first, and it is the *left* parietal bone, which is that part of the head which is deepest in the pelvis:

"During the further progress of labour, viz. whilst the head is pressing through the superior aperture of the pelvis and descending into the pelvic cavity, the two fontanelles are frequently on a level with each other; sometimes the anterior one sinks deeper down, but more frequently the posterior one, and the sagittal suture continues parallel with the right oblique diameter of the superior aperture and cavity of the pelvis. If the head has already descended into the pelvic cavity, and begins to experience the resistance which the inclined plane formed by the lower half of the sacrum, the coccyx, and sacro-ischiatic ligaments offer to it, it usually makes (and sometimes even earlier) the following turn: the antero-posterior diameter of the head gradually turns from the right oblique diameter of the pelvic cavity into the transverse, and then into the left oblique diameter.

"This turn, as just stated, usually takes place gradually, and in screw-like movements forwards and backwards. If we examine at this time between the pains, and find the posterior fontanelle backwards and to the right, we shall frequently find it during the next pain, especially when the contraction of the uterus is at its height, turned quite to the right side, viz. towards the descending ramus of the right ischium, and as the pain gradually subsides, it returns to its former position. On repeating these examinations during and between the pains, or by keeping the finger in contact with the head, we shall find that the posterior fontanelle which, between the pains is to the right, is turned forwards during the pain towards the right foramen ovale, and returns again somewhat as the pain recedes; or in other words, the head moves with the sagittal suture out of the transverse diameter of the pelvic cavity into the left oblique one, and gradually returns to the transverse diameter again, until at last, at the end of the third stage of labour, the posterior fontanelle remains turned towards the right foramen ovale." (§ 265.)

"As in cases of the first cranial position towards the end of the third stage, the head presses with the posterior and superior quarter of the right parietal bone against the os externum, so in the present instance it is the posterior and superior quarter of the left parietal bone which is turned towards the os externum, and which first advances through it." (§ 266.)

It is to Professor Naegele, senior, that we are indebted for having first pointed out the remarkable fact that in the position of the head which after the first occurs most frequently, and where the occiput during the latter periods of labour is universally allowed to be turned forwards, and to the right, the posterior fontanelle is *originally* directed towards the right sacro-iliac synchondrosis, and *not* to the right foramen ovale as commonly supposed, but that as the head approaches the outlet of the pelvis the occiput turns forwards into this position. The correctness of this interesting fact is now generally recognized over the whole German part of the continent; it was made known in this country fifteen years ago, by the translation of Professor Naegele's essay on the mechanism of parturition; it has been practically established in the Midwifery Reports of the General Lying-in Hospital by the translator Dr. Rigby, and is confirmed and taught by Dr. J. G. Simpson, the present distinguished professor of Edinburgh. So far from the position with the posterior fontanelle to the right and forwards being of such frequent occurrence, as an original position it is one of the rarest. In 3491 cases of cranial position observed by Professor Naegele, jun., it occurred only four times.

The position with the anterior fontanelle forwards, and to the right is also very rare. In the above-mentioned 3491 cases it was observed only eight times. From facts, however, of which we have been for many years aware, we are inclined to think that this proportion is too small, and that the position with the posterior fontanelle to the left sacro-iliac synchondrosis, and the anterior one to the right foramen ovale occurs, at an early period more frequently than is supposed: that, as labour advances the posterior fontanelle turns towards the left foramen ovale, as in the second position it does from right sacro-iliac synchondrosis to the right foramen ovale. This we have ourselves observed, and we suspect that the apparent rarity of the position is rather due to this change taking place sooner during labour than in the other case.

Sometimes also the head, when in the second position, does *not* make the usual turn; the anterior fontanelle, which was originally directed forwards and to the right, instead of turning towards the right sacro-iliac synchondrosis, turns more forwards towards the right foramen ovale, and the superior and anterior quarter of the left parietal and upper portion of the left frontal bone, is the part which first passes through the os externum.

On the subject of face presentations, Professor Naegele gives much valuable information. According to his experience the face may present in two ways, viz.

"1st. With the right half of the face (as being that part which is deepest in the pelvis) presenting. The forehead turned to the left.

"2d. With the left half of the face presenting. The forehead turned to the right." (§271.)

Of these the former is by far the most frequent. According to the observations of Professor Naegele, jun. it occurred 20 times out of 31 cases of face presentations which he carefully noted; the 11 other cases being with the forehead to the right.

"In cases of the first position of the face, on passing the finger through the os uteri at the beginning of labour, we usually touch the nose. If we pass it along the ridge of the nose to the left, we shall reach the frontal suture; to the right, we shall feel the nostrils; and anteriorly, the right eye. The long diameter of the face, viz. from the middle of the forehead to the chin, is therefore more or less in the direction of the transverse diameter of the superior aperture of the pelvis, and the right half of the face is lower than the left.

"As the head sinks deeper into the pelvic cavity, during the further progress of labour, it gradually turns, so that at the end of the third stage we shall find the long diameter of the face in the left oblique diameter of the pelvic cavity, the chin towards the right foramen ovale, and the right cheek to the os externum.

"As the face begins to distend the vagina, so that the right cheek and right angle of the mouth first become visible between the labia, the chin advances beneath the descending ramus of the right pubic bone." (§ 273.)

The livid swelling which the face brings with it into the world, and which corresponds to the caput succedaneum of cranial positions, is situated on the right cheek and right half of the mouth, whereas, on the second position of the face, it occupies the left side.

The fact that presentations of the face have, in all probability, been originally those of the cranium, has not met, we think, with the attention it deserves, since a knowledge of it tends greatly to simplify and explain the phenomena of these presentations. In our very first volume we pointed it out, and may therefore quote our own words in illustration of it.

"When the head presents at the brim of the pelvis in the first position—viz. the occiput forward and to the left—if by any cause the occiput should be prevented descending into the cavity of the pelvis, or the forehead experience unusual facility in entering it, the head will turn upon its transverse diameter, and the forehead, followed by the face, will sweep past the right sacro-iliac synchondrosis, and occupy the pelvic cavity, while the occiput still remains at the brim; in other words, the first position of the head becomes the first position of the face; and the frequency of that face presentation where the chin is turned towards the right side of the pelvis, (first position of the face,) in proportion to those where it corresponds to the left side (second position of the face), tends not a little to confirm this view." (Brit. and For. Med. Rev., vol. I, p. 110.)

According to the experience of Professor Naegele, as there are two positions of the head and two of the face, so also are there two positions of the nates, viz.

"1st. Nates presentations, with the back turned forwards towards the anterior wall of the uterus.

"2d. Nates presentations, with the back backward.

"In both cases the back of the child is usually found directed more or less to one side, the ischia corresponding to one or other of the oblique diameters of the pelvis." (§ 279.)

Of 109 cases of nates presentation which Professor Naegele, jun. carefully observed, 72 were in the first, and 37 in the second position.

Our limits will not permit us to quote Professor Naegele, senior's, description of the manner in which the nates pass through the pelvis during labour, although we feel that we are leaving incomplete the series of quotations which we had intended making on this interesting subject. It is one which will well repay the trouble of closely investigating it; and we can, from ample experience, assure our younger readers that nothing makes a practitioner so at his ease with unnatural cases as an accurate and familiar knowledge of those which are natural.

Professor Naegele describes a very unusual mode in which the head will sometimes pass through the pelvis in cases of nates presentation. Instead of the head being pressed with the chin upon the breast, as is usually the case, the occiput, as in presentations of the face, is pressed against the nape of the neck. The head enters the brim of the pelvis in this position with the occiput in advance, and the vertex turned towards one or other of the iliac bones of the mother; and as it passes into the cavity of the pelvis, the vertex turns into the hollow of the sacrum, so that when the head is coming through the os externum, the vertex passes over the perineum, the under surface of the lower jaw being turned towards the symphisis pubis.

The real cause of danger to the child's life during labour with the feet foremost is admirably set forth by this distinguished author. In no case can so much mischief be done, and that so easily, as in nates or footling presentations. In no case is a thorough knowledge of, and confidence in, the powers of the uterus, and the application of judicious assistance at the right moment, so important. In pointing out the injurious effects of attempting to hurry the expulsion of the child, Professor Naegele well observes:

"If it be the uterus which expels the child by the pressure it exerts upon it in all directions, the chin, firstly, will continue pressed upon the breast during the whole passage of the child through the pelvis, and thus ensures the head passing through in the most favorable position. In the second place, the arms remain pressed upon the breast, and are born with it at the same moment. Thirdly, the soft passages will be sufficiently dilated by the gradual advance of the child in order that they should offer less resistance to the head when it follows; and, fourthly, by the gradual progress of the labour, the uterus is only gradually emptied of its contents, its contractile power increased, and thus enabled, by sufficiently powerful contractions, to force the head into and through the pelvis within the proper time. But if the child be pulled at, whereby the pressure of the uterine contractions, which acts on all sides of the child, and which keeps the chin and arms pressed upon the breast, the arms slip up on each side of the head, the chin recedes from the breast, and the head approaches the brim of the pelvis in the most unfavorable position, together with the arms at the same moment. The soft parts are dilated too quickly and violently, and the contracting power of the uterus much injured from its contents having been too rapidly withdrawn. It therefore becomes unable to force the head into and through the pelvis." (§ 289.)

Professor Naegele, jun., has furnished us with copious literary references on the subject of the treatment and management of a woman during natural labour; but, in other respects, the rules which he has given apply merely to midwives, and, indeed, are nearly those of his father's Hebammenbuch. Among these rules there is one which we must not pass by unnoticed, viz., that of giving an enema at the commencement of every labour—a practice which is much neglected in this country. Besides rendering the labour more cleanly, an enema, by unloading the rectum of a mass of solid feces, is frequently the means of removing a serious obstacle to the passage of the head, of rendering the pains much more regular and effective, and the os uteri more dilatable.

We must venture to differ from our excellent author in saying that it is not necessary to preserve any great degree of regularity in suckling a child during the first six weeks after birth, beyond its being put to the breast every two or three hours, for we consider that the strictest regularity and punctuality in this respect is of the utmost importance to its health. During the day it ought to be nursed every two hours and a half, and on no account ought this rule to be transgressed. If placed in a warm cot it will sleep much better than at the mother's side, for the vicinity of the breast is sure to make it restless, and continually craving for more food. A healthy new-born child may soon be taught to sleep for four, five, or six hours without requiring food, and this is of great importance as regards the comfort of the mother.

We here close our review of Professor Naegele's work for the present, and shall delay any remarks on the second part of it (Dystocia) until this portion of his son's book has appeared.

Although we are fully aware that a feeling of regret has long existed that Professor Naegele, sen. has not favoured the world with a work of higher pretensions than a Lehrbuch for Midwives, and that a more elaborate work founded upon it would be a great desideratum; we cannot but regret that Professor Naegele, jun. has confined himself so strictly to the very paragraphs of his father's work, the more so when we call to mind his own excellent publications on obstetric auscultation (translated by Dr. West), and on the mechanism of parturition, which have given ample proofs of learning, industry, and talent, to entitle us to higher expectations.

Of the work of Professor Naegele, sen. we cannot speak too highly or with too much respect; his searching and accurate analysis of the whole progress of natural labour; his masterly descriptions of the various phenomena connected with it; and admirable arrangement of its different modifications, and particularly of the whole subject of Dystocia, which we hope on some future occasion to bring before our readers, have rendered the profession deeply indebted to him, and placed the name of Naegele in the highest ranks of merit and worth.

---

